# BP1 transcriptionally activates *bcl-2 *and inhibits TNFα-induced cell death in MCF7 breast cancer cells

**DOI:** 10.1186/bcr1766

**Published:** 2007-09-13

**Authors:** Holly S Stevenson, Sidney W Fu, Joseph J Pinzone, Jinguen Rheey, Samuel J Simmens, Patricia E Berg

**Affiliations:** 1 Center for Cancer Research, National Cancer Institute, National Institutes of Health, 37 Convent Drive, Bethesda, MD 20892, USA; 2 Department of Biochemistry and Molecular Biology, George Washington University Medical Center, 2300 Washington, DC 20037, USA; 3 Department of Internal Medicine, The Ohio State University College of Medicine, 1581 Dodd Dive, Columbus, OH 43210, USA; 4 Comprehensive Cancer Center, The Ohio State University Medical Center, 1581 Dodd Drive, Columbus, OH 43210, USA; 5 Department of Epidemiology and Biostatistics, George Washington University Medical Center, 2300 Washington, DC 20037, USA

## Abstract

**Introduction:**

We have previously shown that the Beta Protein 1 (BP1) homeodomain protein is expressed in 81% of invasive ductal breast carcinomas, and that increased BP1 expression correlates with tumor progression. The purpose of our current investigation was to determine whether elevated levels of BP1 in breast cancer cells are associated with increased cell survival.

**Methods:**

Effects on cell viability and apoptosis of MCF7 cells stably overexpressing BP1 were determined using MTT and Annexin V assays, and through examination of caspase activation. TNFα was used to induce apoptosis. The potential regulation of apoptosis-associated genes by BP1 was studied using real-time PCR and western blot analyses. Electrophoretic mobility shift assays, site-directed mutagenesis, and transient assays were performed to specifically characterize the interaction of BP1 with the promoter of the *bcl-2 *gene.

**Results:**

Stable overexpression of BP1 led to inhibition of apoptosis in MCF7 breast cancer cells challenged with TNFα. Increased BP1 resulted in reduced processing and activation of caspase-7, caspase-8, and caspase-9, and inactivation of the caspase substrate Poly(ADP-Ribose) Polymerase (PARP). Increased levels of full-length PARP and a decrease in procaspase-8 were also associated with BP1 overexpression. The *bcl-2 *gene is a direct target of BP1 since: (i) BP1 protein bound to a consensus binding sequence upstream of the *bcl-2 *P1 promoter *in vitro*. (ii) MCF7 cells overexpressing BP1 showed increased levels of *bcl-2 *mRNA and protein. (iii) Transient assays indicated that increased *bcl-2 *promoter activity is due to direct binding and modulation by BP1 protein. BP1 expression also prevented TNFα-mediated downregulation of *bcl-2 *mRNA and protein.

**Conclusion:**

These findings suggest mechanisms by which increased BP1 may impart a survival advantage to breast cancer cells, which could lead to increased resistance to therapeutic agents in patients.

## Introduction

Homeobox genes are an important class of master regulatory genes that encode transcription factors responsible for orchestrating developmental processes in many species of animals, as well as in plants and fungi. These genes are characterized by a conserved 180-nucleotide sequence coding for a 60-amino-acid homeodomain that directs binding to downstream target genes that may be activated or repressed. An increasing number of investigations support the involvement of homeobox genes in tumorigenesis of prostate, lung, renal, ovarian, colorectal, and breast tissues [1,2]. Specifically in breast cancer, altered levels of various homeobox genes are directly associated with cellular transformation, disruption of the cell cycle, apoptosis, and progression to a metastatic phenotype [3-7].

Beta Protein 1 (BP1) belongs to the Distal-less subfamily of the homeobox gene family [8]. BP1 maps to chromosome 17q21-22, a region of DNA that is often amplified in breast cancer and that contains the tumor suppressor gene *BRCA1 *and the oncogene *ErbB2 *[9]. We have found that BP1 is expressed in 81% of invasive ductal breast tumors [10,11]. Notably, BP1 expression correlates with breast cancer progression [11], suggesting BP1 may be important in breast tumorigenesis. We have yet to fully understand, however, the functional consequences of its increased expression. Our earlier studies demonstrated that BP1 is expressed in 63% of acute myeloid leukemias but is not detectable in normal lymphoid cells or in normal bone marrow [12]. In clonogenic assays, K562 erythroleukemia cell lines stably overexpressing BP1 showed a 45-fold increase in the number of cells able to grow in soft agar compared with control cells, but we did not observe differences in cell number per colony [12]. These results indicate that BP1 may play an oncogenic role by increasing cell survival.

Tumor cells are notorious for escaping cell death and often develop resistance to therapeutic agents through activation of antiapoptotic mechanisms. Apoptosis is coordinated by cascades of caspases, a family of cysteine proteases that cleave various substrates, ultimately leading to the destruction of the cell. Two primary pathways of apoptosis have been established. The death-receptor pathway, or extrinsic pathway, is triggered through binding of cytokines (TNFα, TRAIL, Fas ligand) to their respective receptors that belong to the TNF receptor family [13]. The mitochondrial pathway, or intrinsic pathway, is regulated by proapoptotic and antiapoptotic members of the Bcl-2 family, which collectively govern the permeability of the mitochondrial membrane [13,14]. Crosstalk between these two pathways can occur, whereby the mitochondrial pathway is triggered following death receptor activation [15,16].

Our objective in the present investigation was to determine whether BP1 impacts antiapoptotic pathways in breast cancer cells. Specifically, we demonstrate that increased BP1 expression protects MCF7 cells challenged with TNFα, resulting in inhibition of apoptosis. We also show that BP1 protein binds to and directly activates expression of *bcl-2*, an antiapoptotic gene. These findings provide evidence of a role for BP1 in cell survival and define mechanisms by which BP1 expression may be tumorigenic.

## Materials and methods

### Cell culture and generation of stable cell lines

MCF7 cells were transfected with either the empty vector pcDNA3.2 (Invitrogen, Carlsbad, CA, USA) or a plasmid containing the BP1 open reading frame under control of the cytomegalovirus promoter. Plasmid-containing cell lines were selected in 800 μg/ml G418. Cells were maintained in RPMI 1640 supplemented with 10% fetal bovine serum, penicillin/streptomycin, 500 μg/ml G418, and 2 mM glutamine. MTT assays were performed to measure cell viability. Cells were seeded in triplicate in 96-well plates, and were cultured in normal growth media containing 20 ng/ml human TNFα (Sigma-Aldrich, St Louis, MO, USA) or were left untreated. After 72 hours, samples were incubated with 5 mg/ml MTT at 37°C for 4 hours. Formazan crystals were dissolved in dimethylsulfoxide (Sigma-Aldrich). Samples were read at 570 nm with a Versamax microplate reader (Molecular Devices, Sunnyvale, CA, USA).

### Annexin V assay

Cell lines were cultured at 3 × 10^5 ^cells/well in six-well plates, and were cultured in normal growth media containing 20 ng/ml TNFα for 18 hours or were left untreated. Cells were labeled with a 1:100 dilution of Annexin V–FITC conjugate and 5 μg/ml propidium iodide according to the manufacturer's instructions (Trevigen, Gaithersburg, MD, USA). Each sample was analyzed using a Nikon Eclipse TE300 inverted epifluorescence microscope (Nikon Instruments Inc, Melville, NY, USA) with filter sets for FITC and TRITC. Early apoptotic cells were distinguished by the presence of green staining in the plasma membrane and the absence of red nuclear staining.

### Electrophoretic mobility shift assays

Complementary sequences spanning 2,555 to 2,513 nucleotides upstream of the *bcl-2 *ATG start site were annealed and 5'-end-labeled with γ-^32^P-ATP using T4 kinase (Invitrogen). The Wheat Germ Coupled Transcription/Translation kit (Promega, Madison, WI, USA) was used to generate BP1 protein from the plasmid pGEM7 containing the BP1 open reading frame. Unlabeled competitor oligonucleotides were added at 500× or 1,000× molar excess to binding reactions. For supershift analyses, binding reactions included BP1 antibody [8]. The following sequences were used as probes and competitors: *bcl-2*, 5'-ACGGTGGGCCTGAAAGTTACTATATGGAAGTCCTCATCGTGTA-3'; mutant *bcl-2*, 5'-ACGGTGGGCCTGAAAGTTAGCTCGACGAAGTCCTCATCGTGTA-3'; negative control, 5'-TCTTAGAGGGAGGGCTGAGGGTTTGAAGTCCAACTCCTAAGCC-3'.

### Luciferase reporter assays

A construct containing the *bcl-2 *P1 promoter region linked to a luciferase reporter gene (LB170) was a kind gift from Dr Linda Boxer (Stanford University, Stanford, CA, USA). Cells were transfected with 2.5 μg LB170 and 1 μg plasmid encoding β-galactosidase, using Fugene 6 Transfection Reagent (Roche, Indianapolis, IN, USA) at a 3:2 ratio of Fugene:DNA according to the manufacturer's instructions. Forty-eight hours post transfection, β-galactosidase activity was measured using the Beta-Galactosidase Enzyme Assay System (Promega), and the luciferase reporter activity was assayed using the Luciferase Assay System (Promega). Luciferase activity output was given in relative light units. The relative light unit value for each sample was divided by the β-galactosidase activity to normalize differences in transfection efficiencies. Each transfection was performed three times in duplicate.

### Site-directed mutagenesis

Using LB170 as a template, mutation of the BP1 binding site was performed using the Quik Change II XL Site-Directed Mutagenesis kit (Stratagene, La Jolla, CA, USA). HPLC-purified complementary primers (Invitrogen) were designed to delete a seven-nucleotide region of the BP1 consensus binding site (underlined): 5'-'GGTGGGCCTGAAAGT TACTATATGGAAGTCCTCATCGTGTA-3'. Plasmids containing the deletion were designated delLB170. Subsequently, using delLB170 as the template, plasmids were generated to contain the mutant BP1 binding site (GCTCGAC), and were designated mutLB170.

### Reverse transcription and quantitative PCR

Total RNA was extracted using Trizol Reagent (Invitrogen) according to the manufacturer's instructions. Reverse transcription of mRNA was performed using the iScript cDNA Synthesis Kit (Biorad, Hercules, CA, USA). TaqMan analyses of BP1 and 18S were performed using QPCR Master Mix Plus reagent (Eurogentec, San Diego, CA, USA). For SYBR Green analyses of *bcl-2*, the reactions were performed using iTaq SYBR Green Supermix with ROX (Biorad). The cycling conditions were as follows using the ABI Prism 7000 Sequence Detection System (Applied Biosystems, Foster City, CA, USA): 50°C for 2 minutes, then 95°C for 10 minutes, followed by 40 cycles at 95°C for 15 seconds and 60°C for 1 minute. SYBR Green analyses also included a dissociation protocol.

The ABI Prism software was used to perform an automatic cycle threshold analysis and to generate a standard curve for extrapolation of the sample data. Mean values of each gene were normalized to the corresponding mean value for 18S. The following sequences were used for primers and probes: 18S primers, 5'-GCCGCTAGAGGTGAAATTCTTG-3' and 5'-CATT CTTGGCAAATGCTTTCG-3'; 18S probe, 5'-ACCGGCGCAAGACGGACCAG-3'; BP1 primers, 5'-CCTCCCCCAATTTGTCCTACTC-3' and 5'-GGTTGCTGGCAGGACAGGTA-3'; BP1 probe, 5'-AGCCAGCGAACCCCGGAGACTC-3'; *bcl-2 *primers, 5'-TGGGATGCCTTTGTGGAACT-3' and 5'-GAGACAGCCAGGAGAAATCAAAC-3'.

### Western blot analysis

Cell lysates were prepared in ice-cold RIPA lysis buffer (50 mM Tris, pH 7.5, 2 mM ethylenediamine teraacetic acid, 100 mM NaCl, 1% NP-40) containing 1× Complete Mini protease inhibitor cocktail (Roche). Proteins were separated by SDS-PAGE and were transferred to a polyvinylidene difluoride membrane. Blots were probed overnight at 4°C with rabbit anti-BP1 (Novus Biologicals, Littleton, CO, USA) at a 1:5,000 dilution, or with a 1:1,000 dilution of mouse anticaspase-7 and anticaspase-8 antibody, rabbit anticaspase-9 and anti-PARP (anti-Poly(ADP-Ribose) Polymerase) antibody (Cell Signaling, Danvers, MA, USA) or mouse anti-*Bcl-2 *antibody (Santa Cruz, Santa Cruz, CA, USA). After washing, blots were incubated with either horseradish peroxidase-linked goat anti-mouse (1:500 dilution) or donkey anti-rabbit secondary antibodies (1:15,000 dilution). Signals were detected using SuperSignal West Dura Extended Duration Substrate (Pierce, Rockford, IL, USA). Relative band intensities were quantitated using the Kodak Image Station 2000 MM and the Kodak ID software (version 3.6.4; Scientific Imaging System, Eastman Kodak Co., Rochester, NY, USA) and by standardizing protein levels against β-actin.

### Statistical methods

Statistical tests comparing mean levels were performed with SAS software based on *a priori *analysis of variance contrasts. Each replicate was treated as an independent observation. Except where noted, contrasts involving MCF7/EV cells were based on averaging across EV1 and EV2. Luciferase values were log-transformed and the percentage of positive cells stained with Annexin V was arcsine-transformed for significance testing. Results are declared significant at α = 0.02, two-sided.

## Results

### BP1 inhibits TNFα-mediated cell death through a caspase-dependent mechanism

**Figure 1:**
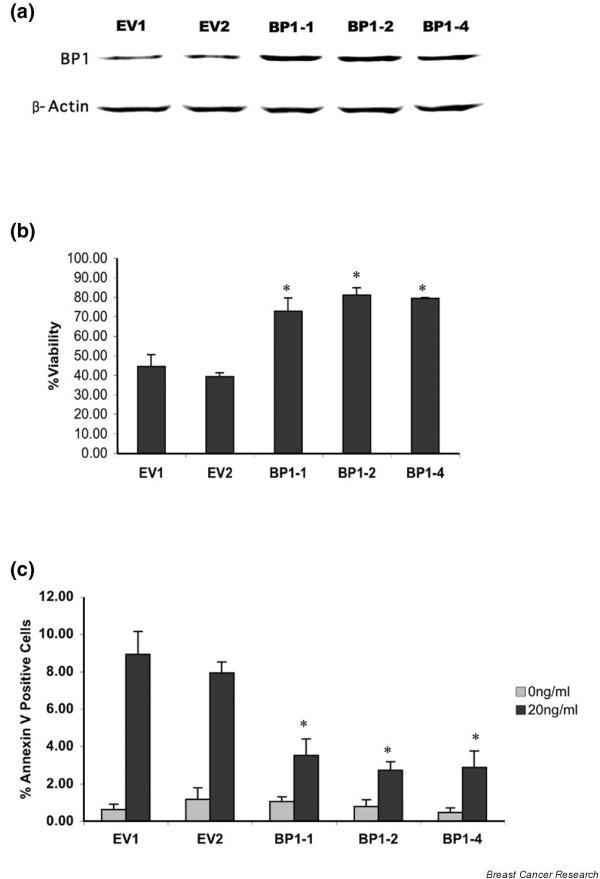
Effect of BP1 on TNFα-induced cell death. **(a) **Western blot analysis of Beta Protein 1 (BP1) protein expression in MCF7/EV cell lines and MCF7/BP1 cell lines. **(b) **MCF7/EV and MCF7/BP1 cell lines were treated with 20 ng/ml TNFα for 72 hours. MTT assays were performed to assess cell viability. To calculate the percentage viability, absorbance values were compared in treated cells versus untreated cells. *Statistically significant differences (*P *< 0.0001). **(c) **MCF7/EV and MCF7/BP1 cell lines were treated with 20 ng/ml TNFα for 18 hours. Cells were labeled with both an Annexin V–FITC conjugate and propidium iodide to distinguish early apoptotic cells. Five fields of cells were photographed and counted for each sample in three independent experiments. The percentage of cells in each field with Annexin V staining in the plasma membrane, but showing exclusion of propidium iodide, is reported. *P < 0.0001.

Three MCF7 cell lines were generated that stably express increased levels of BP1 protein (MCF7/BP1-1, MCF7/BP1-2, and MCF7/BP1-4), as well as two control cell lines containing the empty vector (MCF7/EV1 and MCF7/EV2) (Figure 1a). We first compared the viability of MCF7/EV and MCF7/BP1 cell lines that were grown in the presence or absence of TNFα. As shown in Figure 1b, an average of 43% of MCF7/EV cells survived 3 days post TNFα treatment, whereas all three BP1-overexpressing cell lines displayed an approximately twofold increase in viability (74%, 90%, and 80% for BP1-1, BP1-2, and BP1-4, respectively; *P *< 0.0001 for each comparison). Furthermore, MCF7/BP1 cells exposed to TNFα showed a twofold to threefold decrease in Annexin V binding compared with MCF7/EV cell lines (Figure 1c, *P *< 0.0001 for all three overexpressing cell lines), indicating that increased BP1 expression decreases the ability of MCF7 cells to undergo apoptosis.

**Figure 2:**
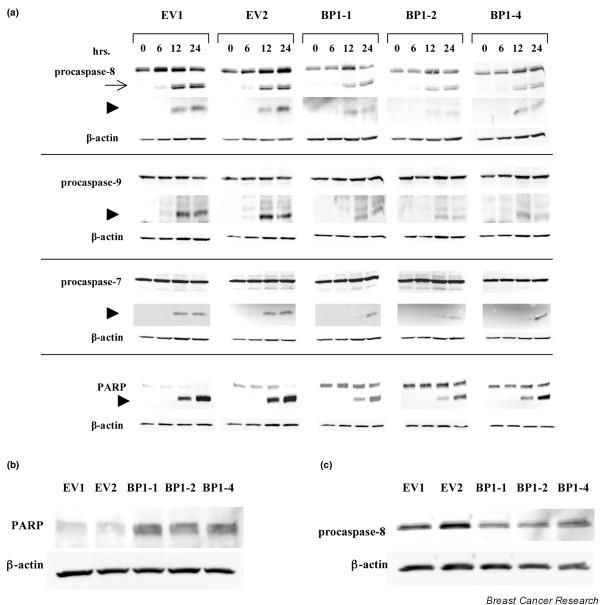
BP1 inhibits TNFα-mediated caspase activation. **(a) **MCF7/EV and MCF7/Beta Protein 1 (BP1) cells were treated with 20 ng/ml TNFα. At the indicated times, protein was analyzed by western blot to examine the expression levels and processing of caspase-8, caspase-9, and caspase-7, as well as the substrate Poly(ADP-Ribose) Polymerase (PARP). In each case, the top band represents the uncleaved, inactive procaspase or full-length active PARP. Arrow, intermediate fragments; arrowheads, position of the expected cleaved product. **(b) **and **(c) **Western blot analyses of PARP and procaspase-8 expression in MCF7/EV and MCF7/BP1 cell lines.

We then examined whether constitutive BP1 expression affected TNFα-mediated cell death through modulation of caspase pathways. Upstream initiator caspase-8 and caspase-9, as well as the downstream effector caspase-7 and its substrate PARP, were analyzed by Western blot analysis in MCF7/EV and MCF7/BP1 cell lines treated with TNFα for various times (Figure 2a). MCF7 cells are deficient in caspase-3 due to a genomic deletion in exon 3 [17], so this caspase was not examined. Processed fragments of each caspase are readily apparent after 12 and 24 hours of exposure to TNFα. Across each cell line, processing of PARP is also seen by 12 hours, with the amount of cleaved protein accumulating through 24 hours. In each MCF7/BP1 cell line, however, there is a clear reduction in cleavage of every caspase as well as of PARP. Analyses of band intensities of each fragment revealed a ≥50% decrease in caspase and PARP cleavage in cells overexpressing BP1, relative to the levels of cleaved products in MCF7/EV cells. Strikingly, untreated MCF7/BP1 cells showed a threefold to fourfold increase in levels of full-length PARP relative to MCF7/EV cells (Figure 2b). In addition, MCF7/BP1 cells show a 1.6-fold to 2.0-fold downregulation of procaspase-8 (Figure 2c), indicating that BP1 may affect the early stages of apoptosis. Together, our findings demonstrate a role for BP1 in caspase-dependent pathways of TNFα-mediated cell death.

### BP1 regulates the expression of Bcl-2

We next sought to define transcriptional targets of BP1 that might explain why its overexpression results in increased cell viability in the presence of TNFα. *bcl-2*, a well-established antiapoptotic oncogene, is often associated with resistance to various cell-death-inducing agents [18]. The *bcl-2 *gene contains two promoters: P1, located 1,386 to 1,423 bp upstream of the translational start site; and P2, located 1.3 kb downstream of P1 [19]. The sequence 5'-TACTATATG-3' matches a consensus binding site for BP1 protein [8] and is located upstream of the P1 promoter at -2539 bp relative to the ATG translational start site.

**Figure 3:**
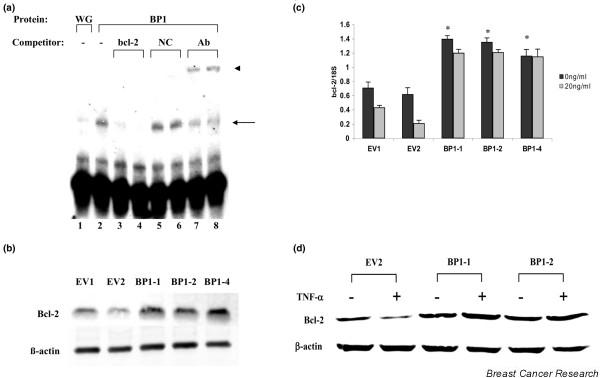
Identification of *bcl-2 *as a putative target gene of BP1. **(a) **Electrophoretic mobility shift assays were performed to detect potential binding of *in vitro *transcribed and translated Beta Protein 1 (BP1) to a consensus binding sequence located in *bcl-2*. Binding of BP1 to a ^32^P end-labeled dsDNA probe containing the putative BP1 binding site and surrounding sequence is observed as a shifted band (arrow). 500× and 1,000× molar excess unlabeled probes or a negative control (NC) sequence lacking a BP1 binding site were added as a cold competitor for BP1 binding. Addition of 1 or 2 μl BP1 antibody (Ab) resulted in a supershift of the original band (arrowhead). Wheat germ extract alone (WG) served as a control. **(b) **Western blot analysis of Bcl-2 protein expression in MCF7/EV and MCF7/BP1 cell lines. **(c) ***bcl-2 *mRNA from each cell line was analyzed by real-time PCR. MCF7/EV and MCF7/BP1 cells were cultured in the presence or absence of TNFα for 72 hours. Data shown represent *bcl-2 *levels normalized to 18S. *P < 0.0001. **(d) **Western blot analysis after culture of cell lines in the presence or absence of TNFα for 72 hours.

An electrophoretic mobility shift assay was used to demonstrate that BP1 protein can specifically bind to a dsDNA oligonucleotide probe containing this site (Figure 3a). A shifted band was observed in the presence of *in vitro *transcribed and translated BP1 protein (lane 2), while a faint band was observed at this location when wheatgerm extract alone was mixed with the *bcl-2 *probe (lane 1). Specificity of the interaction was evidenced by the loss of the shifted band upon addition of 500× or 1,000× molar excess of competitor DNA of the same sequence as the *bcl-2 *probe (lanes 3 and 4). Addition of excess negative control DNA that lacks a BP1 binding site did not reduce the intensity of the band (lanes 5 and 6). In the presence of anti-BP1 antibody we observed both a decrease in the shifted band as well as the appearance of a supershifted band (arrow, lanes 7 and 8), verifying that BP1 protein bound to the *bcl-2 *probe DNA. These data indicate that the *bcl-2 *gene is a potential target for regulation by BP1.

In support of this finding, a comparison of *bcl-2 *expression levels in MCF7/EV and MCF7/BP1 cells by western blot analysis and by real-time PCR revealed a twofold increase in both *bcl-2 *protein (Figure 3b) and mRNA (Figure 3c, black bars; *P *< 0.0001 comparing the average of untreated EV1 and EV2 with BP1-1, BP1-2, and BP1-4).

Constitutive expression of *bcl-2 *abrogates cell death in MCF7 cells exposed to TNFα [20,21]. To examine whether regulation of *bcl-2 *by BP1 is associated with the observed increase in MCF7/BP1 cell viability, *bcl-2 *mRNA expression was analyzed in TNFα-treated cells. Although *bcl-2 *mRNA was downregulated by TNFα in MCF7/EV cells (Figure 3c, gray bars; *P *= 0.004 and *P *< 0.0001 for EV1 and EV2, respectively), BP1-overexpressing cells showed no significant change in *bcl-2 *mRNA after treatment. Consistent with these data, Bcl-2 protein levels are not reduced by TNFα treatment, in contrast to the empty vector control (Figure 3d).

### BP1 directly targets the *bcl-2 *promoter

**Figure 4:**
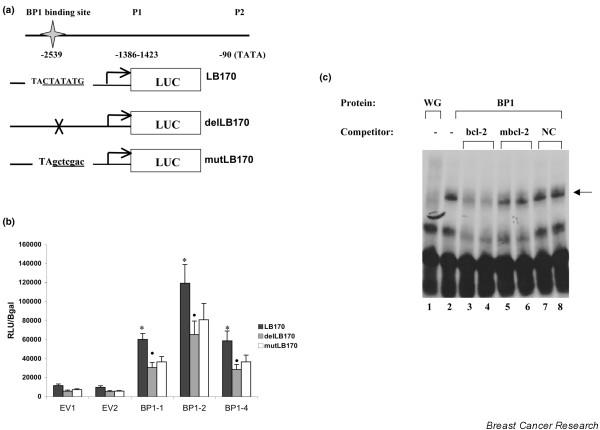
*bcl-2 *is a direct transcriptional target of BP1. **(a) **Schematic diagram of *bcl-2 *P1 promoter constructs. LB170 contains the Beta Protein 1 (BP1) binding site (underlined). delLB170 contains a deletion of seven of the nine nucleotides of the binding site (indicated by X), and mutLB170 contains the mutated BP1 binding site (lowercase, underlined). LUC, luciferase. (b) MCF7/EV and MCF7/BP1 cells were transiently transfected with LB170, delLB170, or mutLB170, as well as a plasmid encoding β-galactosidase. Forty-eight hours post transfection, protein was extracted and assayed for luciferase activity. Relative light units were normalized to β-galactosidase expression units to signify levels of *bcl-2 *P1 promoter activity (RLU/Bgal). *P < 0.0001, ^• ^*P *< 0.05. **(c) **Electrophoretic mobility shift assay: *in vitro *transcribed and translated BP1 protein (BP1) was incubated with a ^32^P end-labeled DNA oligonucleotide probe containing the sequence from the *bcl-2 *promoter including the BP1 binding site (arrow). Cold competitor DNAs including a *bcl-2 *sequence identical to the probe (bcl-2), mutated *bcl-2 *(mbcl-2), and a negative control (NC) lacking the BP1 binding site, were added at 500× or 1,000× molar excess. Wheat germ extract (WG) incubated with the *bcl-2 *probe served as a control.

We next determined whether increased levels of *bcl-2 *expression in MCF7/BP1 cells could be attributed to direct regulation of the *bcl-2 *gene by BP1 protein. A schematic diagram of the promoter region of *bcl-2 *is shown in Figure 4a. MCF7/EV and MCF7/BP1 cell lines were transfected with LB170 (a gift from Dr Linda Boxer, Stanford University), a construct containing the *bcl-2 *P1 promoter region and the 5'-flanking sequence [22], including the BP1 binding site, linked to the luciferase reporter gene. MCF7/BP1-1 and MCF7/BP1-4 consistently showed a fivefold activation of the P1 promoter, whereas MCF7/BP1-2 showed up to an 11-fold increase, compared with levels seen in MCF7/EV control cells (Figure 4b, black bars; *P *< 0.0001 for all three overexpressing cell lines). These results show that BP1 overexpression increased transcriptional activation through the *bcl-2 *promoter. The results do not, however, distinguish between a direct effect, caused by binding of BP1 protein to the promoter, and an indirect effect by BP1, due to regulation of other factors that bind and activate transcription of *bcl-2*. Site-directed mutagenesis and deletion of the BP1 consensus binding site were carried out to differentiate these possibilities.

Using the LB170 construct as a template, a two-step site-directed mutagenesis procedure was performed. First, seven nucleotides of the nine-nucleotide sequence in the BP1 binding site were deleted to generate delLB170, followed by insertion of the mutated sequence, described in [23], to create mutLB170 (Figure 4a). An electrophoretic mobility shift assay was performed to determine whether this mutation could inhibit binding of BP1 to *bcl-2 *(Figure 4c). As before, BP1 protein (WG/BP1) bound to the *bcl-2 *probe, as indicated by the shifted band (lane 2, arrow). No protein binding to the *bcl-2 *probe was observed at this location using the wheatgerm extract (lane 1). Competition with 500× and 1,000× molar excess of unlabeled probe DNA (*bcl-2*) resulted in the loss of the shifted band signal (lanes 3 and 4). If excess competitor DNA containing a seven-nucleotide mutation of the BP1 binding site was added (mbcl-2), however, little competition for binding was observed (lanes 5 and 6). A negative control DNA also did not compete for binding (lanes 7 and 8). This mutation is thus sufficient to disrupt binding of BP1 protein to DNA.

MCF7/EV and MCF7/BP1 cell lines were then transiently transfected with the wild-type LB170, delLB170, or mutLB170. Notably, deletion of the BP1 binding site resulted in an average 45% to 51% decrease in *bcl-2 *promoter activation across all cell lines (Figure 4b, grey bars; *P *< 0.05). Mutation of this site caused an average 37% to 49% reduction in activation of the *bcl-2 *promoter, which was statistically significant for BP1-1 (white bars, *P *= 0.02) but not for BP1-2 or BP1-4, perhaps due to residual BP1 binding to the mutant site (Figure 4c). We thus conclude that BP1 protein can bind to the *bcl-2 *promoter and directly contribute to activation of its expression in MCF7 cells.

## Discussion

Inhibition of apoptosis is a key step in tumor development and growth, promoting the selection and propagation of cells that can resist destruction by various cellular stresses. Evasion of apoptosis by tumor cells has been attributed to downregulation or inactivation of tumor suppressor genes, and to increased activation or expression of oncogenic factors [24]. The studies presented here reveal that high-level BP1 expression is associated with enhanced survival of breast cancer cells challenged with TNFα. Potential mechanisms by which BP1 promotes continued cell viability were identified, involving genes in both extrinsic and intrinsic apoptotic pathways. Specifically, we demonstrated that BP1 can activate *bcl-2 *and PARP, and can repress procaspase-8. BP1 transcriptionally activates *bcl-2 *through direct binding upstream of the P1 promoter region, resulting in a twofold increase in Bcl-2 protein.

Upon either deletion or mutation of the BP1 binding site, we observed an approximately 40% to 50% decrease in *bcl-2 *promoter activity. One possible reason for the remaining activity is that the mutation did not completely prevent BP1 binding. Another possibility is that there may be other factors present that promote *bcl-2 *expression independent of BP1 binding. The plasmid LB170, used in our studies of the *bcl-2 *promoter, contains several binding sites for known transcriptional regulators of *bcl-2*, including Wilms' Tumor 1, SP1, and cAMP response element binding proteins. Wilms' Tumor 1 protein has been associated with aggressive phenotypes of breast cancer and was recently shown to upregulate *bcl-2 *expression in BT-474 breast cancer cells [25]. Additionally, SP1 sites and a cAMP response element are necessary for estradiol-induced *bcl-2 *gene expression in MCF7 and T47D cells [26].

Furthermore, high BP1 expression prevents TNFα-induced downregulation of *bcl-2 *mRNA and protein. This is consistent with data from other laboratories demonstrating that high expression of Bcl-2 promotes cell survival in the presence of TNFα [20,21]. These results not only support our observation that *bcl-2 *is a transcriptional target of BP1, but identify the upregulation of *bcl-2 *as a probable mechanism by which BP1 inhibits cell death.

As mentioned, our previous findings demonstrate BP1 expression in 100% of estrogen-receptor-alpha-negative breast cancers studied, compared with 73% of estrogen-receptor-alpha-positive tumors [11]. This raises the intriguing possibility that BP1 protein and estrogen receptor alpha protein may interact and modulate bcl-2 gene expression and action. There is consequently a possibility that a more robust interaction occurs between BP1 protein and bcl-2 in the absence of estrogen receptor alpha; hence, this would provide an interesting area for future study.

**Figure 5:**
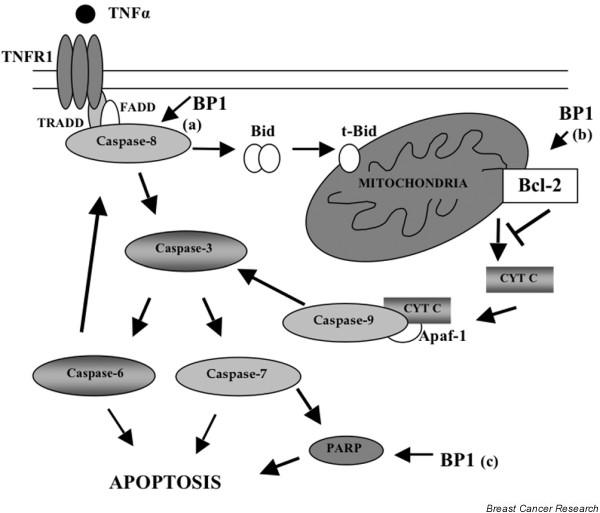
Model for BP1-mediated disruption of caspase activation after TNFα treatment. Arrows indicate the three points (a, b and c) at which Beta Protein 1 (BP1) may impact apoptosis. Bid, BH3 interacting domain death agonist; CYT C, cytochrome c; FADD, Fas (TNFRSF6)-associated via death domain; PARP, Poly(ADP-Ribose) Polymerase; t-Bid, truncated Bid; TNFR1, TNF receptor 1; TRADD, TNFRSF1A-associated via death domain.

Our data further point to a role for BP1 in modulation of caspase-dependent pathways in apoptosis. Increased expression of BP1 reduced TNFα-induced processing of caspase-7, caspase-8, caspase-9, and the caspase substrate PARP by approximately 50%, consistent with the ability of BP1 to enhance cell viability by twofold. These findings suggest a model by which BP1 may modulate TNFα-induced cell death at several points (Figure 5).

First, full-length procaspase-8 expression is decreased in MCF7/BP1 cell lines; lower levels of procaspase-8 may result in less available activated caspase-8, which would lead to decreased activation of downstream caspases and PARP, as we observed. Scanning of the caspase-8 DNA sequence has revealed possible binding sites for BP1 protein, indicating that caspase-8 is a potential transcriptional target of BP1.

Second, Bcl-2 controls the release of cytochrome c from the mitochondria. Following cytochrome c release, crosstalk between the death-receptor and mitochondrial pathways of apoptosis can lead to additional processing of caspase-8 mediated by effector caspases-3 and -6 [27,28]. Owing to its regulation of *bcl-2*, BP1 may reduce activation of those caspases downstream of the mitochondria [29].

A third point at which BP1 may affect apoptosis is through regulation of PARP. We discovered increased levels of full-length PARP in MCF7/BP1 cells. PARP has been shown to be overexpressed in 57% of breast tumors [30]. PARP has multiple roles in cell death, and in regulation of gene expression, proliferation, and differentiation, and is well known for its ability to mediate DNA repair in response to DNA damage [31]. Of relevance here, PARP inhibitors, when used in conjunction with chemotherapeutic drugs or radiotherapy, are known to increase the cytotoxic effects of these agents in tumor cells [32]. We have located potential binding sites for BP1 protein in the PARP genomic sequence, suggesting that PARP is also a possible target gene for regulation by BP1.

## Conclusion

Our findings reveal details of a role for BP1 in caspase-dependent and *bcl-2*-linked mechanisms of tumor cell survival, and suggest BP1 could serve as a marker for drug resistance and a therapeutic target. This is the first study to define a function for increased BP1 expression in breast cancer and to highlight pathways important for further exploration.

## Abbreviations

bp = base pairs; BP1 = Beta Protein 1; FITC = Fluorescein Isothiocyanate; HPLC = high-performance liquid chromatography; MTT = thiazolyl blue tetrazolium bromide; PARP = Poly(ADP-Ribose) Polymerase; PCR = polymerase chain reaction; TNF = tumor necrosis factor.

## Competing interests

George Washington University holds the patent for BP1 antibody, and Novus licenses the technology from George Washington University. The Novus BP1 antibody was used in this research. PEB has previously acted as a consultant for Novus, but is no longer doing so.

## Authors' contributions

HSS carried out the experimental procedures, participated in the design of the study, and drafted the manuscript as part of her PhD requirements at the George Washington University Medical Center. SWF generated the stable cell lines used in the study. JJP assisted in the design of the study and in performing the viability assays. JR carried out western blot analyses of BP1 and SJS performed the statistical analyses. PEB conceived of the study, directed its design and coordination, and helped to draft the manuscript. All authors read and approved the final manuscript.
